# Discriminative features of immunoglobulin G4-related disease (IgG4-RD) and associated autoimmune rheumatic diseases (ARDs) in a nationwide observational cohort: study from the Egyptian College of Rheumatology

**DOI:** 10.1007/s10067-024-07274-y

**Published:** 2025-01-03

**Authors:** Hany El-Saadany, Hanan El-Saadany, Samar Tharwat, Wael Soliman, Shereen El Shereef, Abdelazeim Elhefny, Ahmed Yehia, Emad El-Shebini, Noha Khalil, Aya El-Hindawy, Nevin Hammam, Ahmed El-Saman, Fatma Ali, Shereen Elwan, Tamer A. Gheita, On behalf of the Egyptian College of Rheumatology IgG4 Study group

**Affiliations:** 1 Internal Medicine and Rheumatology Department, Armed Forces College of Medicine, Cairo, Egypt; 2 Rheumatology Department, Faculty of Medicine, Tanta University, Tanta, Egypt; 3 Rheumatology and Immunology Unit, Internal Medicine Department, Mansoura University, Mansoura, Egypt; 4 Department of Internal Medicine, Faculty of Medicine, Horus University, New Damietta, Egypt; 5 Tropical Medicine Department, Faculty of Medicine, Minia University, Minia, Egypt; 6 Rheumatology Department, Faculty of Medicine, Alexandria University, Alexandria, Egypt; 7 Internal Medicine and Rheumatology Department, Faculty of Medicine, Ain Shams University, Cairo, Egypt; 8 Internal Medicine and Rheumatology Department, Faculty of Medicine, Beni Suef University, Beni Suef, Egypt; 9 Internal Medicine and Rheumatology Department, Faculty of Medicine, Menoufia University, Shebin El-Kom, Egypt; 10 Internal Medicine and Rheumatology Department, Faculty of Medicine, Cairo University, Cairo, Egypt; 11 Rheumatology Department, Faculty of Medicine, Cairo University, Cairo, Egypt; 12 Rheumatology Department, Faculty of Medicine, Assiut University, Assiut, Egypt; 13 Rheumatology Department, Faculty of Medicine, Sohag University, Sohag, Egypt; 14 Rheumatology Department, Faculty of Medicine, Minia University, Minia, Egypt; 15 Mansoura University Hospital, El Gomhouria St, Mansoura, 35511 Dakahlia Governorate Egypt

**Keywords:** Autoimmune rheumatic diseases, Immunoglobulin G4-related disease (IgG4-RD), Rheumatological dilemma

## Abstract

**Objective:**

The objective of this study is to present the clinical characteristics of immunoglobulin G4-related diseases (IgG4-RD) patients and describe associated overlap with autoimmune rheumatic diseases (ARDs).

**Patients and methods:**

This cross-sectional study included 81 patients with IgG4-RD who were recruited from 13 specialized rheumatology departments and centers across the country in collaboration with the Egyptian College of Rheumatology (ECR). Patients underwent a thorough history-taking and clinical examination. We reviewed patients’ medical records and recorded the medications they used. The presence of comorbidities or cumulative manifestations was determined. Laboratory investigations, imaging, and biopsy histopathology were assessed.

**Results:**

The mean (SD) age was 41.4 (14.6) years with 60 females and 21 males (F/M 2.9:1). The diagnosis was definite in 50 (61.7%), probable in 19 (23.5%), and possible in 12 (14.8%). The most common cumulative clinical features are IgG4-related respiratory disease in 19 (23.5%), autoimmune pancreatitis (AIP) in 18 (22.2%), and Riedel’s thyroiditis in 17 (21.0%). Approximately 80% were administered corticosteroids, whereas 40% received azathioprine as adjunct therapy. Approximately 16% developed a relapse with this combination and transitioned to an alternative steroid-sparing treatment. Twelve individuals (14.7%) required rituximab. Fifty percent of patients receiving rituximab (six patients) exhibited complete improvement, while the remaining had partial improvement. Ten (12.3%) patients had associated ARDs: five (6.2%) with systemic lupus erythematosus (SLE), four (4.9%) with rheumatoid arthritis (RA), and one with vasculitis. Of the four patients with associated RA, three were rheumatoid factor (RF) negative. IgG4 was in all cases, RF was positive in 18.5%, and antinuclear antibody was in 14.7%.

**Conclusion:**

IgG4-RDs exhibit a wide range of presentations, closely associated with ARDs. Awareness among clinicians about this condition will increase their consideration and rate of prompt diagnosis, which is essential to prevent damage to critical organs.

**Table:** 

Key Points
• *IgG4-RDs have a myriad spectrum of presentation with a close link to rheumatic diseases*. • *Awareness among clinicians about this condition will increase their consideration and rate of prompt diagnosis*. • *The lack of reliable biomarkers for this condition has been an important hurdle for diagnosis*.

## Introduction

Immunoglobulin G4-related disease (IgG4-RD) is a systemic fibroinflammatory disorder that can impact several organ systems [1]. There is increasing evidence that the pathogenesis of IgG4-RD has an autoimmune basis [2]. It is a condition that is characterized by increased serum concentrations of IgG4 antibodies as well as infiltration of plasmacytes that express IgG4 in the organs affected [3]. Currently, serum IgG4 represents one of the most important biomarkers for IgG4-RD, and it is used for both the diagnosis and the monitoring of one’s response to treatment [2]. IgG4 is a subclass of immunoglobulin G. Serum IgG4 levels may be a reflection of the activity and severity of the disease, and its level exhibits a positive correlation with the number of organs that are affected. Its role in inflammation is still being determined, as the significance of its anti-inflammatory activity and tolerance-inducing properties is counteracted by its pathogenic features exhibited in IgG4-RD [4].

IgG4-RD is an emerging condition that is increasingly being diagnosed due to improvements in clinical awareness. This disease affects both men and women, and it can affect multiple organs, most commonly the salivary and lacrimal glands, as well as the pancreas and liver [5]. Large-vessel vasculitis is a well-known feature of IgG4-RD [1]. Coronary arteritis and periarteritis are significant manifestations of IgG4-RD, making its recognition as a variable-vessel vasculitis probable [1]. Aortitis, too, may be unusually associated with IgG4-RD [6]. Moreover, IgG4-related cerebral vasculitis rarely exists and may pass unnoticed [7].

The diagnosis and management of IgG4-RD remain challenging as biomarkers and therapies are being investigated. Hallmark features on biopsy are the gold standard to confirm a diagnosis, while serum IgG4 level confers a smaller contribution [5]. The diagnosis of IgG4-RD is primarily based on histopathological findings, but when vascular involvement occurs, imaging modalities such as magnetic resonance imaging (MRI) and PET are similarly important because they help in mapping the disease and identifying other affected organs that are more accessible to biopsy [8]. Critical histopathological features are a dense lymphoplasmacytic infiltrate, a storiform pattern of fibrosis, and obliterative phlebitis, and its diagnosis is based primarily on the morphological appearance on biopsy, while tissue IgG4 counts and IgG4/IgG ratios are secondary in importance [9]. An attempt has been made to construct a valid predictive index for IgG4-RD [10], and further work on this disease is encouraged and warranted. Steroids are the most effective initial management for this condition, with limited effectiveness as maintenance therapy [5]. IgG4-RD is a fibroinflammatory disorder that swiftly responds to B-cell depletion therapy with rituximab (RTX) [11].

Mimickers of IgG4-RD include autoimmune diseases such as vasculitis, sarcoidosis, Sjogren’s syndrome (SS), and inflammatory bowel disease [5]. IgG4-RD with multiple organ involvement may be suspected of having systemic lupus erythematosus (SLE) [12]. Cases with polymyositis and elevated serum IgG4 levels and IgG4 plasma cells in the muscles may mimic IgG4-RD [13]. Clinicians should pay attention to the development of IgG4-RD and vasculitis throughout the long course of other autoimmune disorders such as Hashimoto’s thyroiditis [14] and increased IgG4 levels have been found in several autoimmune diseases, including primary SS, systemic sclerosis (SSc), and SLE [15]. The distinguishing of IgG4-RD from other autoimmune rheumatic diseases (ARDs) has not been well-examined and remains challenging. Therefore, the aim of the present study was to present the clinical characteristics of IgG4-RD patients and to describe the associated overlap with ARDs presented to the rheumatologist.

## Patients and methods

This cross-sectional study included 81 IgG4-RD patients fulfilling the 2011 comprehensive diagnostic criteria [16] and/or the 2019 American College of Rheumatology/European League Against Rheumatism (ACR/EULAR) classification criteria [17]. They were recruited from 13 specialized rheumatology departments and centers representing 12 major governorates all over the country in collaboration with the Egyptian College of Rheumatology (ECR) during the period between January 2022 and May 2023. Patients in the corresponding university-teaching hospitals and centers of excellence provided informed consent to participate in accordance with the 1964 Helsinki Declaration and its later amendments [18]. The study was approved by the Mansoura Faculty of Medicine Institutional Research Board (MFM-IRB) (approval number: R.23.09.2349).

Patients were subjected to full history-taking and clinical examination. The presence of co-morbidities or cumulative manifestations was determined based on the information documented in the medical records. The medications used to treat IgG4-RD, and the responses were documented. The complete blood count (CBC), erythrocyte sedimentation rate (ESR), C-reactive protein (CRP), total serum IgG and IgG4, presence of rheumatoid factor (RF) and antinuclear antibody (ANA) positivity, and complements level (C3 and C4) were determined. In addition, patients were assessed, and their clinical and laboratory data were amended to account for the presence of co-existing ARD, provided that the diagnosis had been approved by a rheumatology specialist.

Contrast-enhanced computed tomography (CECT), Positron emission tomography and computed tomography (PET-CT), ultrasound, and magnetic resonance imaging (MRI) were done as required. Tissue biopsy and histopathology were performed accordingly and included the number of IgG4 + plasma cells per high-power field (HPF) identified by immunohistochemical staining. A biopsy showing greater than 10 IgG4 + plasma cells per HPF is considered positive. In 2011, a consensus of Japanese experts proposed the comprehensive criteria to classify IgG4-RD as “definitive,” “probable,” or “possible” based on a combination of clinical, serological, and pathological features. In particular, a “possible” diagnosis of IgG4-RD is established in the absence of pathological confirmation [16].

### Statistical analysis

Data was collected and analyzed on a standardized data sheet and stored in an electronic database. Variables were analyzed as observed, with no imputation of missing data. Statistical Package for Social Sciences (SPSS) version 25 was utilized. Variables were presented as frequencies and percentages or means and standard deviations. A comparison between groups was done using the Chi-square test, Mann–Whitney *U* tests, or analysis of variance (ANOVA), as an appropriate *p*-value less than 0.05 was considered significant.

## Results

### Sociodemographic data and clinical characteristics

The current study included 81 IgG4-RD patients with a mean age of 41.4 ± 14.6 years. They were 60 females and 21 males (F/M 2.9:1). The characteristics of patients are presented in Table 1. The diagnosis was definite in 50 (61.7%), probable in 19 (23.5%), and possible in 12 (14.8%). The most common cumulative clinical features are in the following order: IgG4-related respiratory disease in 19 (23.5%), autoimmune pancreatitis (AIP) in 18 (22.2%), Riedel’s thyroiditis in 17 (21.0%), and IgG4-related renal disease and IgG4-related liver disease in 16 (19.8%) and 15 (18.5%), respectively. One patient also had dry eyes and mouth but with negative anti-Ro and anti-La. Exophthalmos and atrial mass were present in one patient each, ascites in two, obstructive uropathy in two, and hematemesis in six. One patient had papillary thyroid carcinoma, and another had a large midline brain temporal lobe cyst. Two patients were reported to have coronavirus disease 2019 (COVID-19) associated with immunosuppression. Ten (12.3%) patients had associated ARDs, five (6.2%) with SLE, 4 (4.9%) with rheumatoid arthritis (RA), and one with vasculitis. Of the four patients with associated RA, three were RF-seronegative.

**Table 1:** Characteristics of the immunoglobulin G4-related disease (IgG4-RD) patients

Parameter mean ± SD, IQR, or *n* (%)	IgG4-RD patients (*n* = 81)
Age (years)	41.4 ± 14.6
F/M	60:21 (2.9:1)
Disease duration (mon)	49.03 ± 37.7
Age at onset (years)	42.7 ± 12.4
BMI	28.7 ± 20.5
Smoking	6 (7.4)
*Co-morbidities*	
Diabetes	12 (14.8)
Hypertension	14 (17.3)
Thyroid disease	13 (16)
*Associated ARD*	10 (12.3)
SLE	5 (6.2)
RA	4 (4.9)
Vasculitis	1 (1.2)
Organs involved (*n*)	2 (1–3)
IgG4-related respiratory disease	19 (23.5)
Autoimmune pancreatitis (AIP)	18 (22.2)
Riedel’s thyroiditis	17 (21)
IgG4-related renal disease	16 (19.8)
IgG4-related liver disease	15 (18.5)
Inflammatory orbital pseudotumor	12 (14.8)
Sclerosing cholangitis	11 (13.6)
Retroperitoneal fibrosis	11 (13.6)
Lymphoplasmacytic gastritis with AIP	10 (12.3)
Mikulicz disease	8 (9.9)
Chronic sclerosing dacryoadenitis	7 (8.6)
IgG4-related mesenteritis	7 (8.6)
Chronic sclerosing aortitis/periaortitis	5 (6.2)
Ovary disease	4 (4.9)
Nasopharynx	3 (3.7)
Sclerosing sialadenitis	3 (3.7)
IgG4-related hypophysitis	3 (3.7)
IgG4-related pachymeningitis	3 (3.7)
Midline destructive disease	3 (3.7)
Prostatitis	3 (3.7)
Constrictive pericarditis	1 (1.2)
*Investigations*	
ESR (mm/1st hr)	46.02 ± 29.6
CRP (mg/dl)	20.7 ± 20.5
Consumed C3	4 (4.9)
Consumed C4	5 (6.2)
IgG total (mg/dL)	2647.49 (990–4450)
Serum IgG4 (mg/dL)	410.32 (165–986)
High IgE	17 (21)
High IgG4	81 (100)
Positive RF	15 (18.5)
Positive ANA	12 (14.8)

*IgG4-RD* immunoglobulin G4-related disease, *ARD* autoimmune rheumatic diseases, *BMI* body mass index, *ESR* erythrocyte sedimentation rate, *CRP* C-reactive protein, *C* complement, *RF* rheumatoid factor, *ANA* antinuclear antibody, Mikulicz disease (dacryoadenitis and sialadenitis)

Table 2 delineates the administered immunosuppressive agents and the corresponding therapeutic response. Approximately 80% were administered corticosteroids, whereas 40% received azathioprine as adjunct therapy. Approximately 16% developed a relapse with this combination and transitioned to an alternative steroid-sparing treatment. Twelve individuals (14.7%) required rituximab. Fifty percent of patients receiving rituximab (6 patients) exhibited complete improvement, while the remaining had partial improvement.

**Table 2:** Medications received by the immunoglobulin G4-related disease (IgG4-RD) patients and response to treatment

Treatment *n* (%)	IgG4-RD patients (*n* = 81)
*Medical*	
Steroids	63 (77.7)
Azathioprine	33 (40.7)
Rituximab	12 (14.8)
Mycophenolate mofetil	12 (14.8)
Colchicine	10 (12.3)
Cyclophosphamide	3 (3.7)
Methotrexate	2 (2.5)
Cyclosporine	1 (1.2)
Improvement on steroids	33 (40.7)
*Complete remission*	26 (32.1)
*Partial remission*	7 (8.6)
Improvement on cDMARDs	26 (32.1)
Improvement on rituximab	6 (7.4)
Surgical stenting of CBD	4 (4.9)

*IgG4-RD* immunoglobulin G4-related disease, *cDMARDs* conventional disease-modifying anti-rheumatic drugs, *CBD* common bile duct

### Radiological data

**Fig. 1:**
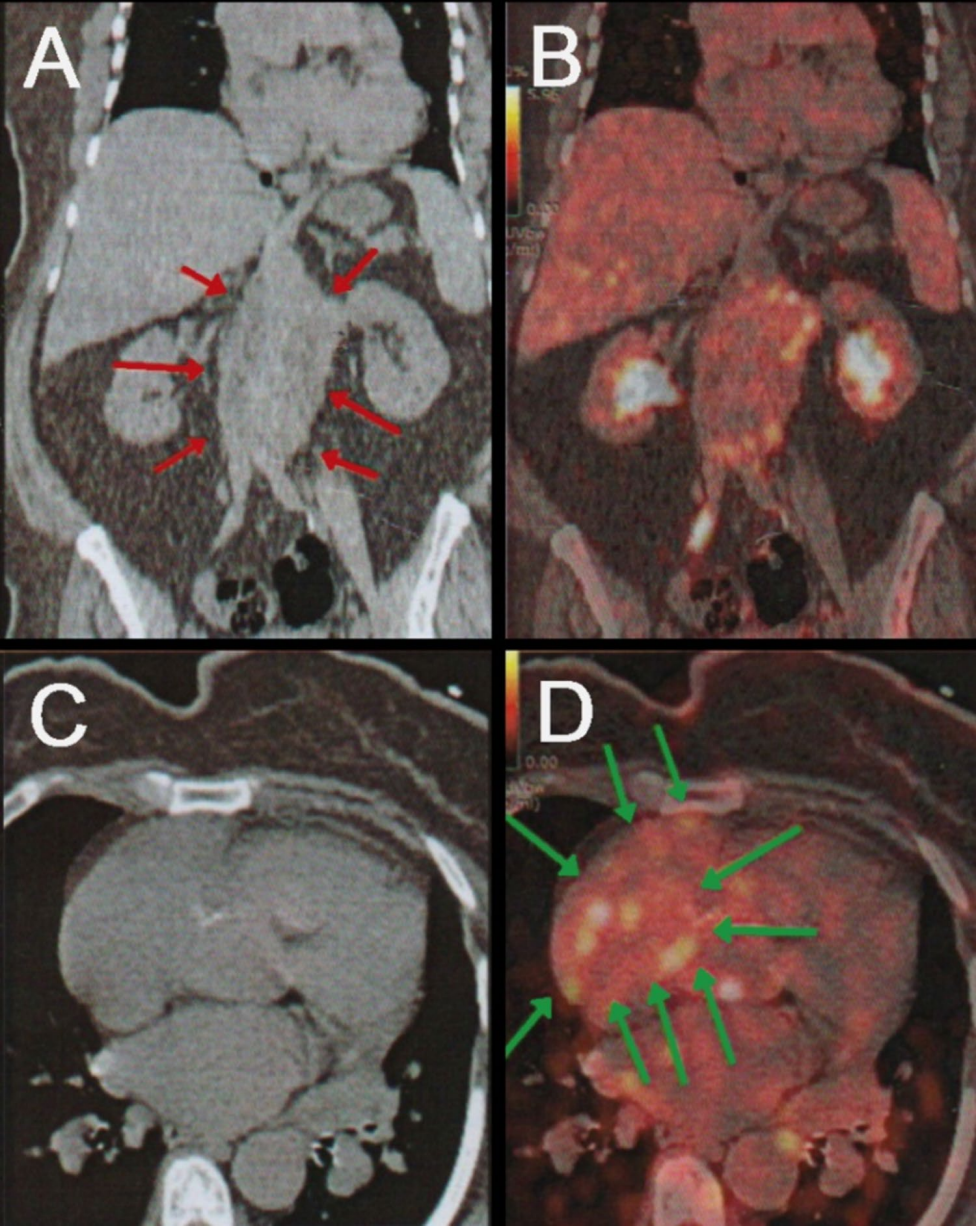
A 48-year-old female with IgG4-related disease. **A** Coronal non-contrast-enhanced computerized tomography (CT) and **B** coronal 18 fluorodeoxyglucose (FDG)-positron emission tomography (PET)/CT fusion images show 18 FDG-avid retroperitoneal soft tissue mass of amalgamated lymph nodes encasing the abdominal aorta and its branches up to the iliac bifurcation (red arrows). **C** Trans axial non-contrast-enhanced CT and **D** trans axial FDG PET/CT fusion images show 18 FDG-avid pericardial soft tissue mass compressing the right atrium (green arrows)

On CECT, the delayed and “rim” enhancement was present in 11 patients and the irregular pancreatic duct in five. Abdominal ultrasound revealed hepatomegaly in 15 patients, splenomegaly in four, an enlarged pancreas in five, cholecystitis, thyroiditis, and an atrophic kidney in one patient each. MRI revealed pancreatitis in two, common bile duct stricture in two, retroperitoneal fibrosis (RPF) in one, orbital pseudotumor in one, and brain edema in one. PET CT detected increased uptake in the orbit of two patients, metabolically active peri-aortic and RT atrium masses in one patient (Fig. 1), a prominent left lacrimal gland in another case, a mesenteric lesion with enhancement uptake in another case, and a superior mesenteric vein thrombosis in another.

### Tissue biopsy histopathology

Tissue biopsy was done in 61 (75.3%) shows the following: IgG-4-positive plasma cells (≥ 10 IgG-4-positive plasma cells per HPF) present in 25 (30.9%), dense lymphoplasmacytic infiltrate with obliterative fibrosis was present in 47 (58%), dense lymphocytic infiltrate was present in two (2.5%), storiform fibrosis in 40 (49.4%), obliterative phlebitis in 27 (33.3%), and un-informative biopsy was present in two (2.5%).

The patients were categorized as follows: definite IgG4-RD (61.7%), characterized by clinical symptoms, elevated IgG4 levels, and pathological features; probable (23.5%), based on clinical examination and histological findings; and possible (14.8%), determined by clinical findings and elevated serum IgG4 levels.

## Discussion

This study elucidates the clinical aspects of IgG4-RD patients who presented to rheumatology clinics and provides evidence that IgG4-RD might manifest concomitantly with other ARDs.IgG4-RD has a diverse range of clinical manifestations that are closely associated with ARDs [19]. It is crucial to raise physicians’ awareness of this disease to boost their level of concern, which is necessary for preventing damage to essential organs.

IgG4-related disease is a clinical entity first described in Japan and includes a wide range of conditions, including Mikulicz’s disease (MD), autoimmune pancreatitis (AIP), interstitial nephritis, prostatitis, and retroperitoneal fibrosis. The IgG4-related disease, an immune-mediated fibroinflammatory condition that affects multiple organs, can lead to tissue-destructive lesions and organ failure [19]. Vascular involvement (thickening) in the setting of IgG4-RD is increasingly recognized in the form of aortitis with possible aneurysm formation. Other affected vessels like iliac, coronary, and carotid arteries are often underdiagnosed [8]. There is an ongoing demanding need to develop comprehensive diagnostic criteria for IgG-RD as cases are increasingly reported worldwide, provided the difficulty of acquiring biopsy samples, the sensitivity and specificity of the cut-off level of serum IgG4, and impaired immunostaining of IgG4 [19]. The natural history and prognosis of IgG4-RD have not been sufficiently defined. Even though spontaneous improvement occurs, the disease often recurs without treatment. Most patients initially and effectively respond to steroids, but relapses are inevitable on discontinuation, and some cases require the addition of RTX. Significant organ dysfunction may result from uncontrolled and increasing inflammatory and fibrotic changes in the afflicted tissues. The elevated risk of malignancy with IgG4-RD requires further research [20, 21].

IgG4 plays an important role in autoimmune rheumatic diseases, yet its role in the course and pathogenesis of ARDs is still not fully understood. Increased IgG4 levels have been reported in rheumatoid arthritis (RA), although no definite link with disease activity has been identified. The potential importance of IgG4 concentrations, both elevated and decreased, in SS has been verified. Additionally, significant IgG4 titters have been detected in SLE patients, and it has been confirmed that these antibodies reduce complement consumption and the production of proinflammatory cytokines, thus effectively slowing the progression of SLE. The gut microbiome encodes disease-triggering or -sustaining factors in both IgG4-RD and SSc and may account for such overlap [22]. IgG4-RD presents a challenge to physicians’ full balance of skills because of its multi-organized nature and the importance of close clinicopathologic links in disease management. Although IgG4-RD has pathological characteristics strongly suggestive of the diagnosis and a growing number of typical clinical features are now recognized, clinicians who see patients with IgG4-RD are rarely comfortable making the diagnosis without histopathologic confirmation and the exclusion of potentially serious mimics [23].

In the current work, the IgG4-RD cases presented to the rheumatology clinics were mostly females (F/M 2.9:1). On the other hand, IgG4-RD cohorts generally have a slight predominance of middle-aged and older males [24]. In the present work, *ARDs* were present in 12.3% of IgG4-RD cases; 6.2% had associated SLE, 4.9% RA, and 1.2% vasculitis. It is interesting to note that the overlap of SLE and IgG4-RD is quite uncommon; in the literature, there have been just eight patients [25–32] who have presented with such a presentation. Among the patients in our cohort, we found five SLE patients who also had IgG-RD. The characteristics of the present IgG4-RD cases with SLE are compared to those of other cases that have been reported all over the world in Table 3. Rheumatic diseases may involve the pituitary gland, and the spectrum includes IgG4-RD in 45% and SLE and vasculitis in 22% each, with SS representing 11%. Such patients improve on steroids and immunosuppressants but may also require long-term hormone replacement therapy for pituitary disorders [33]. One of the present cases had dry eyes and mouth but with negatively related autoantibodies. IgG4-RD may involve the salivary and lacrimal glands making it important to differentiate from SS. In fact, IgG4-related ocular disease entails aqueous tear deficiency and eye dryness [34]. A recently published study of 234 patients diagnosed with IgG4-RD identified the presence of concomitant systemic rheumatic disease in six patients, accounting for 3% of the cohort. These six patients consisted of an equal distribution of three males and three females and included sarcoidosis, Takayasu arteritis, RA, eosinophilic granulomatosis with polyangiitis (EGPA), and granulomatosis with polyangiitis (GPA) [35]. IIgG4-RD is characterized by the presence of auto-reactive clones of enlarged cytotoxic T-lymphocytes (CTLs) that produce interferon-gamma that have also been observed in various other autoimmune and rheumatic disorders [36, 37]. More research with a larger patient population is needed to determine whether the co-existence of IgG4-RD and other immunological diseases is limited to specific disease phenotypes. Furthermore, it remains to be seen whether this temporal link also underpins a pathogenic correlation and whether there are other immunological abnormalities that predispose to IgG4-RD.

**Table 3:** Cases of IgG4-RD/immunoglobulin G4-related disease (IgG4-RD) with systemic lupus erythematosus

Country	China [26]	Japan [27]	Japan [28]	Japan [29]	Japan [30]	Japan [31]	USA [32]	USA [33]	Egypt (current 5 cases)				
Year	2021	2022	2020	2019	2018	2007	2019	2015	2023	2023	2023	2023	2023
Gender	M	M	M	F	M	F	F	F	F	F	F	F	F
IgG4-RD onset age	67	73	46	58	74	37	63	71	26	21	20	20	18
Initial disease	SLE	sim	SLE	sim	IgG4	sim	sim	sim	SLE	sim	sim	sim	sim
Duration till both(y)	2	sim	8	sim	0.5	sim	3	sim	4	sim	sim	sim	sim
IgG4 (mg/dl)	243		1826	1240		224	452	37	156	170	High	High	165
ANA	+ ve	+ ve	+ ve	+ ve	+ ve	+ ve	+ ve	+ ve	+ ve	+ ve	+ ve	+ ve	+ ve
Anti-dsDNA	+ ve	+ ve	+ ve	+ ve	+ ve	+ ve	+ ve	+ ve	+ ve	+ ve	+ ve	+ ve	+ ve
Antiphospholipid		+ ve											
Consumed C	Yes	-	No	Yes	Yes	Yes	No	No	No	Yes	Yes	Yes	Yes
Comorbidities/associated disease			KFS	None	Gastric cancer	None	HTN; thyroid	None		No	Yes	No	No
Proptosis									Yes	Yes			No
Lacrimal	Yes								Yes	No			No
Retinopathy		Yes											No
Salivary gland	Yes				Yes					No			
Dry mouth	Yes								Yes				
Lymph nodes			Yes	Yes			Yes						
ILD		Yes								No		Yes	No
Pleural effusion					Yes		Yes						
Renal affection	Yes	Yes	Yes	Yes	Yes	Yes	No	Yes	Yes	Yes	Yes	Yes	Yes
CNS			Yes							Yes			Yes
Pancreas						Yes				Yes			Yes
IgG4 biopsy	+ ve	+ ve	+ ve		+ ve	+ ve	+ ve	+ ve	+ ve	+ ve	-	-	-
Treatment	CS; HCQ; MMF	CS; HCQ; BEM	CS; CYC	CS; MMF; BEM	CS	CS	CS; HCQ	CS; MMF	CS; AZA; RTX	AZA; MMF; CsA	CS; MMF	CS; AZA	CS; AZA; RTX
Prognosis	Resolved								Improved				Resolved

*IgG4-RD* immunoglobulin G4-related disease, *sim* simultaneous, *ANA* antinuclear antibodies, *anti-dsDNA* anti double-stranded deoxyribonucleic acid, *C* complement, *ILD* interstitial lung disease, *HTN* hypertension, *KFS* Klinefelter syndrome, *CNS* central nervous system disease, *CORT* corticosteroids, *HCQ* hydroxychloroquine, *MMF* mycophenolate mofetil, *BEM* belimumab, *CYC* cyclophosphamide, *AZA* azathioprine, *RTX* rituximab, *CsA* cyclosporine

All cases had raised IgG4 levels. Elevated IgG4 levels do not confirm, and low IgG4 serum levels do not rule out IgG4-RD. Even patients with mimickers of the disease can have elevated levels [5]. High serum IgG4 levels and IgG4 plasma-cell infiltration in organs are not specific to IgG4-RD [13]. The vital role of the IgG4 ratio to IgE (IgE/IgG4) has been known for years [4]. In ARDs, IgG4 levels were increased in 6.3% of cases and were similar to the normal population, unlike the level in IgG4-RDs [15]. In fact, the elevated levels help diagnose IgG4-RDs and evaluate the therapeutic response to steroids rather than considering the association with another autoimmune disease [38]. On the other hand, serum levels of IgG4 in patients with systemic autoimmune diseases who had not received steroids yet were alike those in IgG4-RD patients with AIP [39].

Rheumatoid factor (RF) was positive in 18.5% of patients, and it was reported to be positive in some patients with IgG4-RD with an unknown role in the disease pathogenesis [11]. An antigen-driven response has been suggested in the generation of IgG4 RF in the RA disease process [40]. Both IgG4 and RF increase the inflammatory response of macrophages caused by immune complexes including anti-citrullinated protein antibodies in the RA synovia [11].

The robust production of type 1 interferon (IFN-I) and IL-33 is well-known in autoimmune disorders, including SLE and psoriasis. Both cytokines are also common in the immunopathogenesis of AIP and IgG4-RD, driven by autoimmunity [3]. High avidity IgG ANAs affect the distribution of ANA IgG3 and IgG4 which play a particular role in the inflammatory process, activity, and therapy of SLE [41]. It was reported that SLE patients had a high frequency of low IgG2, IgG3, IgG4, and IgM levels especially those with lupus nephritis (LN) [42]. Pathognomonic autoantibodies can induce inflammation and tissue injury in SLE. Although IgG4 is mainly non-inflammatory due to its distinctive structure, the role of IgG4 autoantibodies in SLE remains basically unidentified [43]. Interestingly, SLE IgG4 autoantibodies can slow SLE progression by inhibiting complement consumption and inflammatory cytokine production. Novel therapeutic strategies against SLE IgG4 are potentially promising [43].

A few cases of cutaneous vasculitis have been documented in IgG4-RD, all of which exhibited hypocomplementemia, and the role of IgG4 in its etiology remains unknown [44]. MD is one of the IgG4-RDs that affects the cardiovascular system; however, small-sized vasculitis is rarely reported in IgG4-RD [45]. While infrequent, IgG4-RD was found to be associated with medium or small vessel vasculitides, and a new overlap syndrome with ANCA-associated vasculitides (AAV) has recently been reported [46]. Nineteen cases of simultaneous association of AAV and IgG4-RD were described, raising the possibility of an overlap syndrome [46]. The differential diagnosis of these two entities is essential, as AAV necessitates more aggressive immunosuppressive therapy due to its severity and results in a poor outcome, especially with renal involvement [11, 47]. However, high IgG4 positivity may be considered an inflammatory marker of disease severity in the setting of AAV and underlying malignancy, rather than an overlap with IgG4-RD [48]. Furthermore, an unusual overlap of AAV and IgG4-tubulointerstitial nephritis (TIN) with underlying primary SS was also reported [49]. Propylthiouracil used to treat hyperthyroidism was reported to induce an unusual presentation of AAV-associated anti-GBM antibodies, IgA nephropathy, and IgG4 interstitial infiltrate [50]. A co-existing overlap of IgG4-RD with Behçet’s disease has been reported with the existence of a retroperitoneal mass [51]. A final diagnosis of IgG4-RD paved the way for the presence of an atypical laryngeal presentation in a case with AAV and Behçet’s disease [52].

Approaches to the management of IgG4-RD include surgical resection of affected tissues and treatment with steroids, “steroid-sparing” immunosuppressives, or biologics with ongoing efforts to establish formal treatment guidelines [23]. In this work, 14.8% received RTX. In IgG4-RD patients, the protocol of RTX administration is derived from the experience with RA; however, the optimal therapeutic dose required to induce disease remission remains unknown [11]. Surgical stenting of the common bile duct was done in 4.9% of cases.

To the best of our knowledge, little research has been published on IgG4-related disease to evaluate its association with ARDs. This study provides the most recent data on an Egyptian cohort of IgG RD patients. A significant strength of the study was the relatively large sample size. Furthermore, our findings enhance the understanding of epidemiology, hence aiding in the comprehension and management of the disease.

Nevertheless, certain limitations of this study warrant acknowledgment. A primary weakness was the cross-sectional design, which precludes causal inferences. Incorporating ARDs in longitudinal analysis will be essential for elucidating causality. Secondly, there are possible confounders that were not accounted for, including polypharmacy, disease awareness, and drug adherence. Third, we did not examine the types of ARDs that were clustered together, which may have exhibited a distinct prognosis in contrast to isolated disorders or disparate sets of ARDs that were associated concurrently. Nonetheless, it will be addressed in further studies including these populations.

## Conclusion

IgG4-RDs have a myriad spectrum of presentation with a close link to rheumatic diseases. Awareness among clinicians about this condition will increase their consideration and rate of prompt diagnosis, which is essential to prevent damage to critical organs. The lack of reliable biomarkers for this condition has been an important hurdle for diagnosis.

## Data Availability

The datasets used and/or analyzed during the current study are available from the corresponding author upon reasonable request.

## Abbreviations

AAV: ANCA-associated vasculitides
ACR/EULAR: American College of Rheumatology/European League Against Rheumatism
AIP: Autoimmune pancreatitis
ARDs: Autoimmune rheumatic diseases
CBC: Complete blood count
CECT: Contrast-enhanced computed tomography
ECR: Egyptian College of Rheumatology
ESR: Erythrocyte sedimentation rate
FDG-PET: 18F-fluorodeoxyglucose positron emission tomography
IgG4-RD: ImmunoglobulinG4-related disease
IL-4: Interleukin-4
MD: Mikulicz’s disease
MRI: Magnetic resonance imaging
RA: Rheumatoid arthritis
RF: Rheumatoid factor
RPF: Retroperitoneal fibrosis
RTX: Rituximab
SLE: Systemic lupus erythematosus
SS: Sjogren’s syndrome
SSc: Systemic sclerosis
